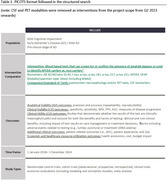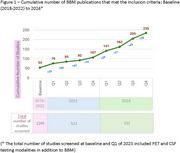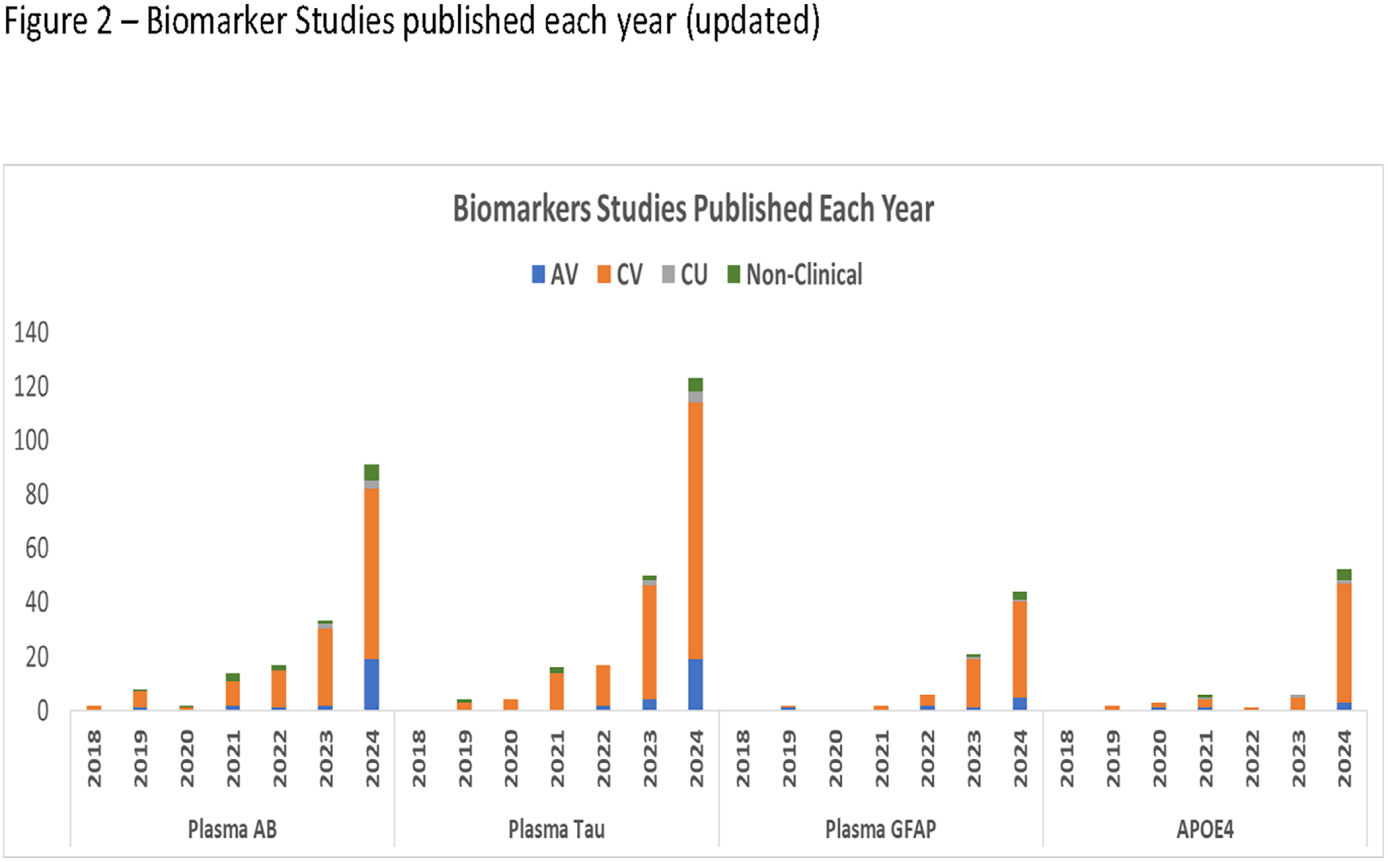# Value of blood‐based biomarker testing to diagnose, identify and monitor patients with Alzheimer's Disease ‐ A structured literature review

**DOI:** 10.1002/alz70856_099842

**Published:** 2025-12-25

**Authors:** Yan Helen Hu, Elizabeth Somers, Miyuru Amarapala, Catheline Plaideau, Miya Strait, Harald Hampel

**Affiliations:** ^1^ Eisai Inc., Nutley, NJ, USA; ^2^ Veranex, Boston, MA, USA

## Abstract

**Background:**

Understanding the available evidence for the validity and utility of tests used to diagnose and monitor patients with early Alzheimer's Disease (AD) is essential for informed care decisions, including amyloid‐targeting monoclonal antibody therapy initiation. While amyloid positron emission tomography (PET) and cerebrospinal fluid (CSF) are considered the current gold standard tests in the US, we see a rapid increase in studies evaluating the use of blood‐based biomarkers (BBM) to identify amyloid pathology in AD. BBM such as β‐amyloid (Aβ), and phosphorylated Tau (including *p*‐Tau 217) offer a more accessible, affordable, and less invasive testing modality for clinical decisions. This study aims to characterize the clinical and non‐clinical evidence of established and emerging AD BBM.

**Method:**

A structured Medline/PubMed search based on the PICOS framework identified full‐text articles published between January 2018 ‐December 2024 reporting clinical and non‐clinical value of AD BBM Studies evaluating analytical validity (AV), clinical validity (CV), clinical utility (CU), or related non‐clinical utility (NCU) parameters of plasma Aβ, Tau, glial fibrillary acidic protein (GFAP) and apolipoprotein E (APOE) biomarkers were retrieved. The target population was adults with mild cognitive impairment or mild dementia due to AD. The quality of evidence was ranked on an adapted Hayes Rating System.

**Result:**

Of the 3559 studies identified, 330 met the inclusion criteria of the structured literature review. The number of publications focused on AD BBM increased exponentially, from 54 during 2018‐2022 to over 200 by the end of 2024. Most studies were focused on the CV of BBMs, while a growing number demonstrated CU and NCU, including the impact of tests on management decisions, cost‐utility, and patient care pathway. There is a continuing interest in ethnically diverse populations and cognitively unimpaired adults at risk of AD.

**Conclusion:**

Increasingly, evidence demonstrates the diagnostic performance of BBM in AD, showing a correlation with PET and CSF as well as clinical utility and economic value. The available evidence supports clinical adoption of BBM in routine patient care including testing patients to select those for targeted AD therapies. Further, payers should facilitate patient access by evaluating reimbursement of BBM for use in AD diagnosis and management.